# Supplementary figures and images for: Rapamycin suppresses inflammation and increases the interaction between p65 and IκBα in rapamycin-induced fatty livers
Source: PLoS One. 2023 Mar 3;18(3):e0281888. doi: 10.1371/journal.pone.0281888 (PMC9983852; doi:10.1371/journal.pone.0281888)

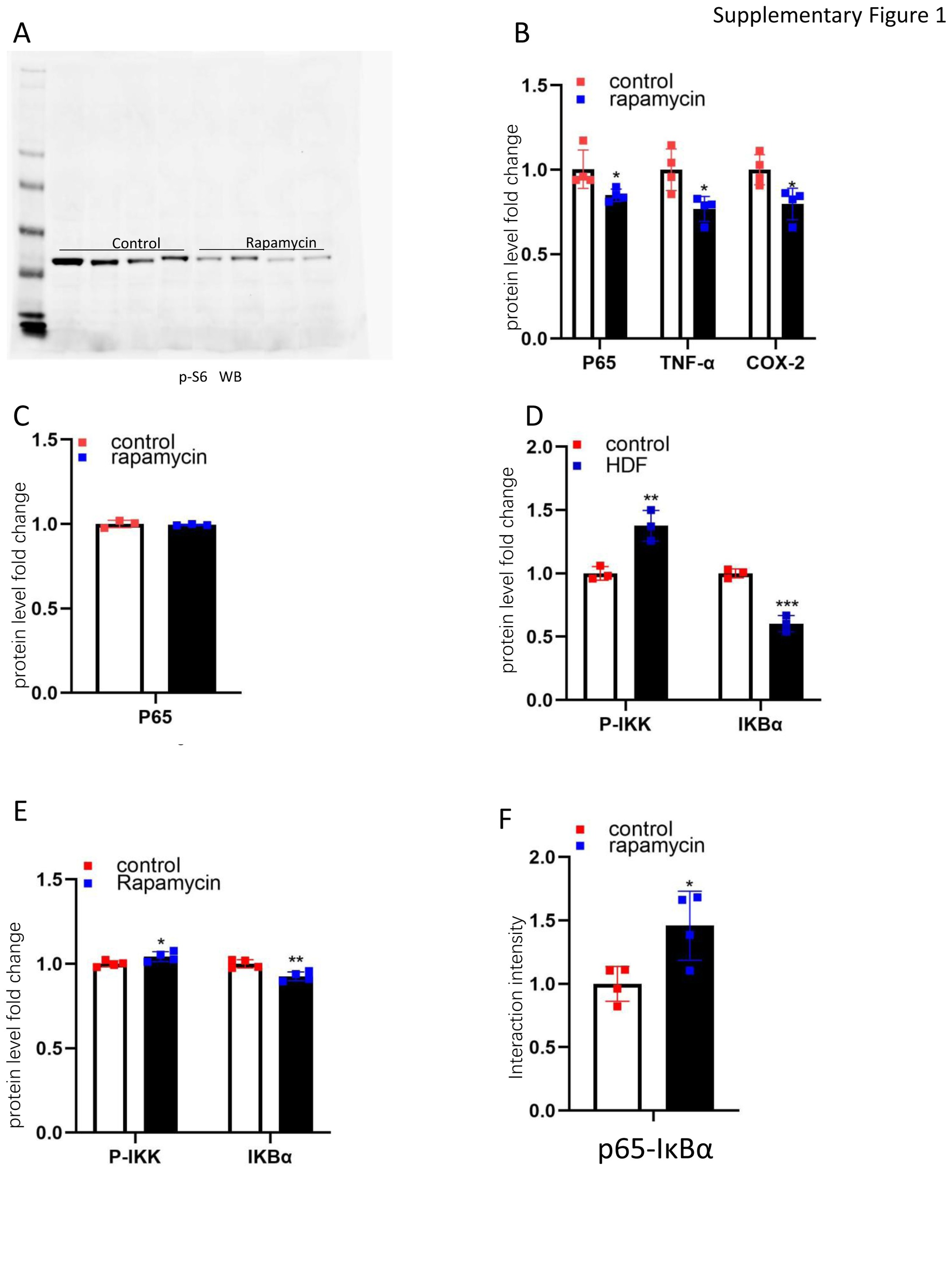

Supplement: S1 Fig — A. Rapamycin decreases the phosphorylation level of S6 protein in mouse livers. B. Quantification of Fig 3A. C. Quantification of Fig 3B. D. Quantification of Fig 3C. E. Quantification of Fig 3D. F. Quantification of Fig 3F. (TIF) [file pone.0281888.s001.tif]

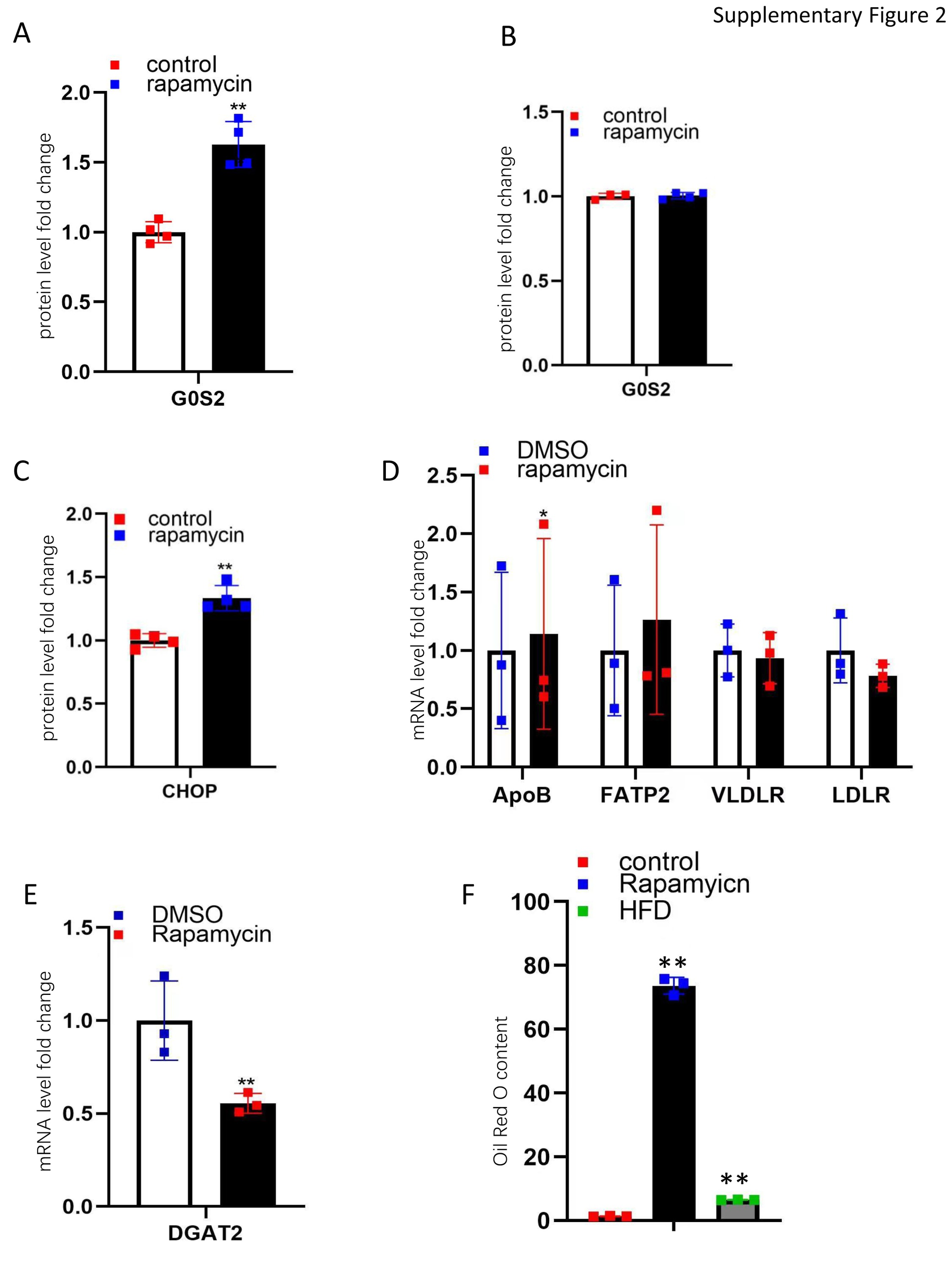

Supplement: S2 Fig — A. Quantification of Fig 4C. B. Quantification of Fig 4D. C. Quantification of Fig 4F. D. mRNA levels of key proteins which may affect lipid transport in livers. E. Rapamycin down-regulates DGAT2 mRNA expression level in mouse livers. F. Quantification of Fig 1B. (TIF) [file pone.0281888.s002.tif]

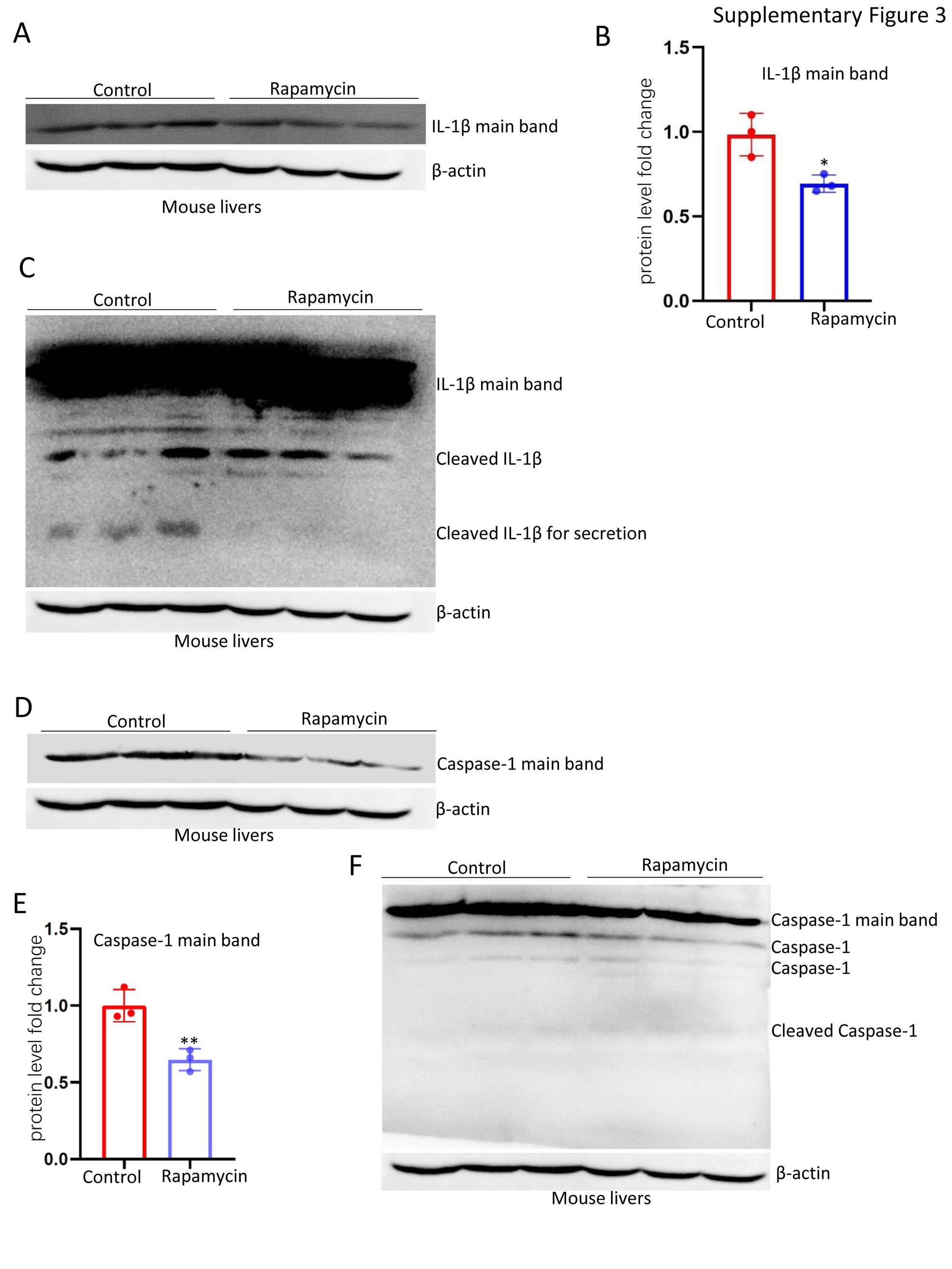

Supplement: S3 Fig — A. IL-1β protein levels were significantly decreased in rapamycin-induced fatty livers (IL-1β main band). B. Quantification of S3A Fig. C. Western blotting showed rapamycin dramatically reduced protein levels of cleaved IL-1β for secretion. D. Caspase-1 protein levels were significantly decreased in rapamycin-induced fatty livers (IL-1β main band). E. Quantification of S3D Fig. Rapamycin effects on protein levels of cleaved Caspase-1. (TIF) [file pone.0281888.s003.tif]
