# Supplementary material for: Rapamycin suppresses inflammation and increases the interaction between p65 and IκBα in rapamycin-induced fatty livers
Source: PLoS One. 2023 Mar 3;18(3):e0281888. doi: 10.1371/journal.pone.0281888 (PMC9983852; doi:10.1371/journal.pone.0281888)
Supplement: S1 Table — (DOCX) [file pone.0281888.s004.docx]

Supplementary Table 1. Mouse qPCR primers

| *Gene name* | *Forward and Reverse primer (5’-3’)* | *Accession number* |
| --- | --- | --- |
| 36B4 | F: AGATTCGGGATATGCTGTTG  R: ACATCACTCAGAATTTCAATGG | NM_007475 |
| HSL | F: TGTGGCTTCTAACCGCAAAAG  R: TGGGCATAGTAGGCCATAGCA | NM_001039507 |
| ATGL | F: TTCACCATCCGCTTGTTG  R: AGTTCCACCTGCTCAGAC | NM_025802 |
| G0S2 | F: GACAGAGAAGGGAGACAC  R: CACAGCAGCAAATCAGTC | NM_008059 |
| CGI-58 | F: CCCTTTCCTTCCAGTATTC  R: CGTAACAGCACCACATAG | NM_026179 |
| ACC1 | F: CAGCAGTTACACCACATAC  R: CCATCTTCATTACCTCAATCTC | NM_133360 |
| SREBP-1c | F: GTAAATCTGCGGGATGATG  R: TGAGTTGGTATCTAGGTCTG | NM_001358314 |
| FASN  DGAT2  ACOX1  CPT-1α  PGC-1α  PPARα  CD36  Mttp  NLRP3  Caspase-1  VDAC2  XBP1s  ATF4  ATF6  BIP  Adamts1  TGF-β1  ApoB  Fatp2  Ldlr  Vldlr | F: TGTGGATGGAGGTATCAAC  R: TGGTAGGCATTCTGTAGTG  F: TGACCACCAGGAACTATATC  R: TGGAGAGCAAGTAGTCTATG  F: GAAATATGCCCAGGTGAAG  R: GTTTATACTGCTGCGTCTG  F: GAGGAGACAGACACCATC  R: TACCGCATCCAGAGATTG  F: GTAAATCTGCGGGATGATG  R: TGAGTTGGTATCTAGGTCTG  F: TTAGAGGAGAGCCAAGTTG  R: ACCGATGGACTGAGAAATC  F: CTGTGTTTGGAGGCATTC  R: ATAACGAACTCTGTATGTGTAAG  F: ATCCTCTTCTGCCTATACTG  R: AATCACCACCTGACTACC  F: ATGCTGGAATTAGACAAC  R: CATTTCACCCAACTGTAGG  F: GCCGTGGAGAGAAACAAGG  R: CAAGTCACAAGACCAGGCATATTC  F: ACCTCGCTTGGACATCAG  R: ACCAGAGCAGACAGTGTAAG  F: GGAAGAAGAGAACCACAAAC  R: ACCAGCCTTACTCCACTC  F: TGGCCAAGCACTTGAAACCT  R: GCCAAGCCATCATCCATAGC  F: CAGTTCAGCCCAAGCCTTTATT  R: TTGCTGGGACACTGGCATT  F: TGCGGCCAAGAACCAACT  R: AGGCGCTTGGCATCGA  F: GCCGGAAGTGACCTCCAAT  R: GGGTGGGTTGTGCTGCTT  F: GCAACAACGCCATCTATG  R: AGGTAACGCCAGGAATTG  F: TTGGCAAACTGCATAGCATCC  R: TCAAATTGGGACTCTCCTTTAGC  F: TCCTCCAAGATGTGCGGTACT  R: TAGGTGAGCGTCTCGTCTCG  F: TGACTCAGACGAACAAGGCTG  R: ATCTAGGCAATCTCGGTCTCC  F: GGCAGCAGGCAATCGAATG  R: GGGCTCGTCACTCCAGTCT | NM_007988  NM_026384  NM_001271898  NM_013495  NM_008904  NM_011144  NM_001159558  NM_001163457  NM_145827  NM_009807  NM_011695  NM_001271730  NM_009716  NM_001081304  NM_022310  NM_006988  NM_011577  NM_009693.2  NM_011978.2  NM_001252658.1  NM_001161420.1 |
