# Supplementary material for: Rapamycin suppresses inflammation and increases the interaction between p65 and IκBα in rapamycin-induced fatty livers
Source: PLoS One. 2023 Mar 3;18(3):e0281888. doi: 10.1371/journal.pone.0281888 (PMC9983852; doi:10.1371/journal.pone.0281888)
Supplement: S1 File — (PDF) [file pone.0281888.s005.pdf]

figure3.A mouse liver

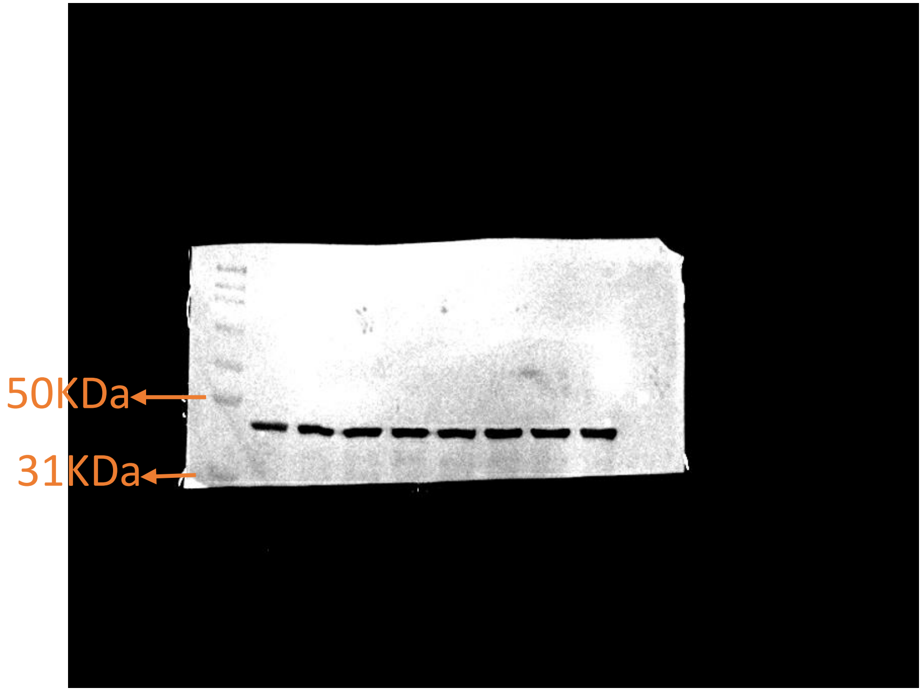


β-actin

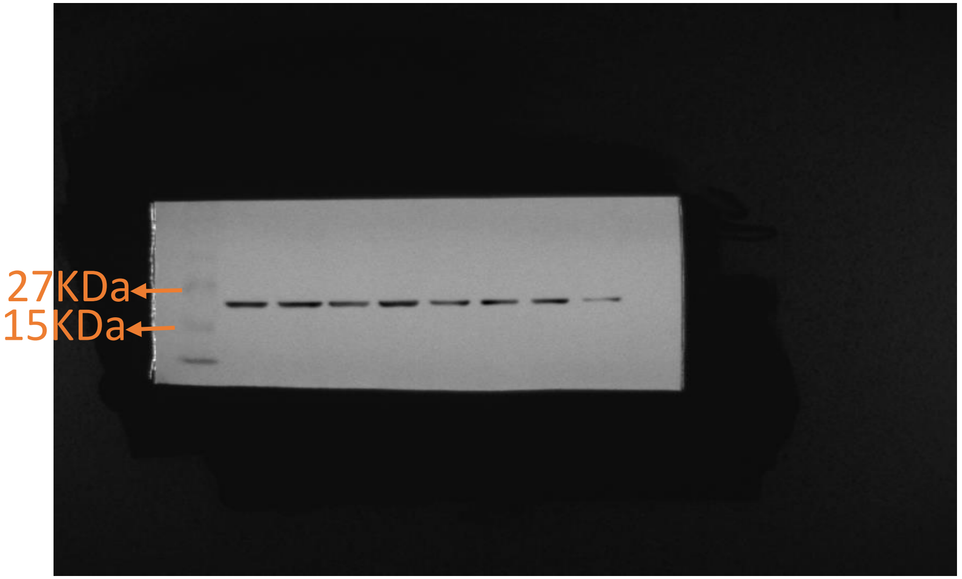


TNFα

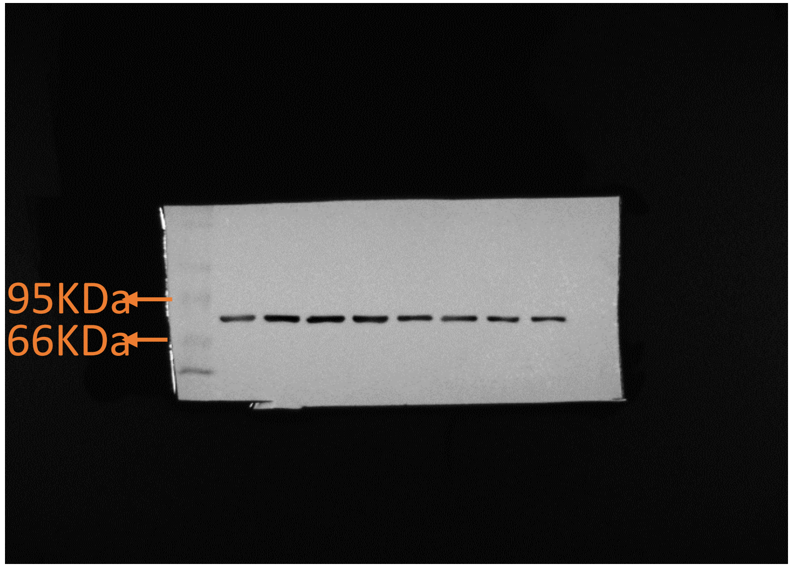


cox2

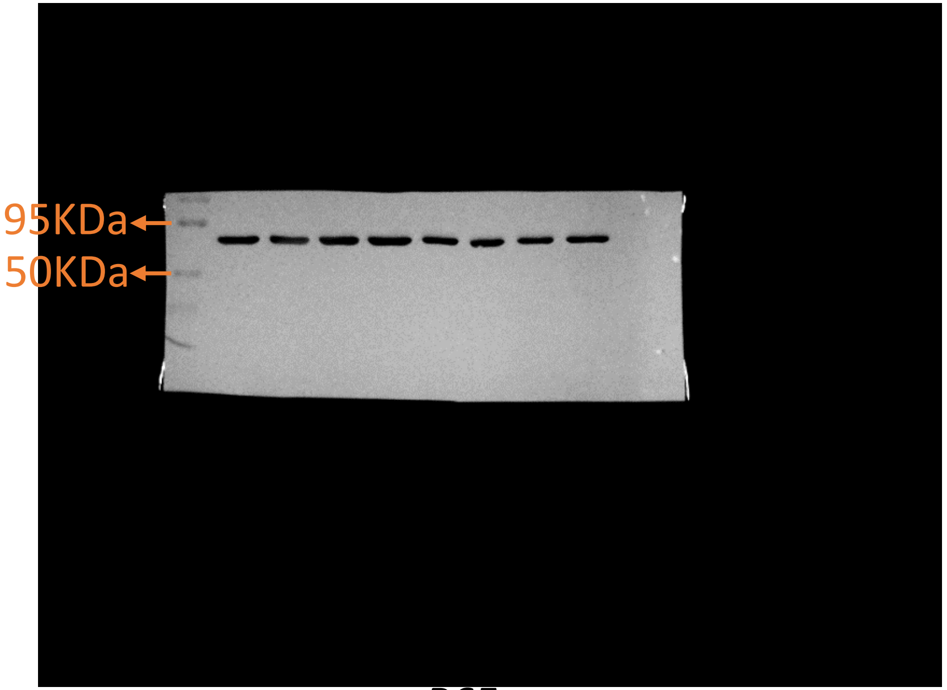


P65

figure3.B mouse primary hepatocytes

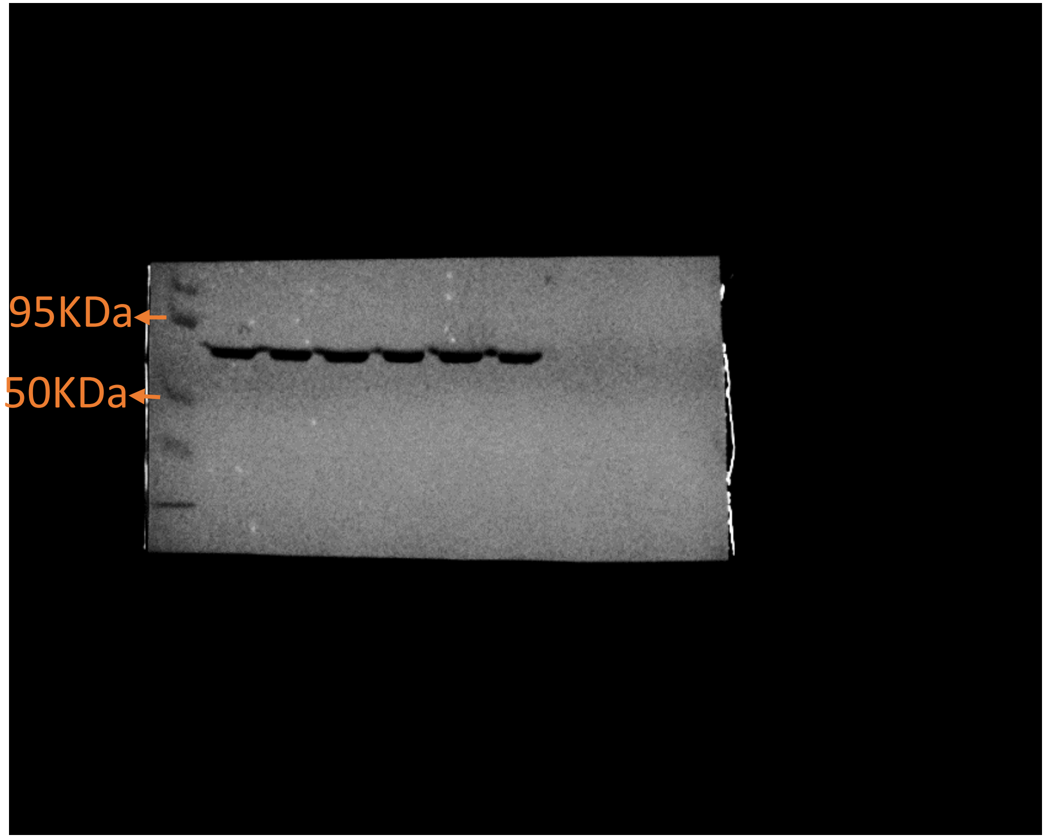


P65

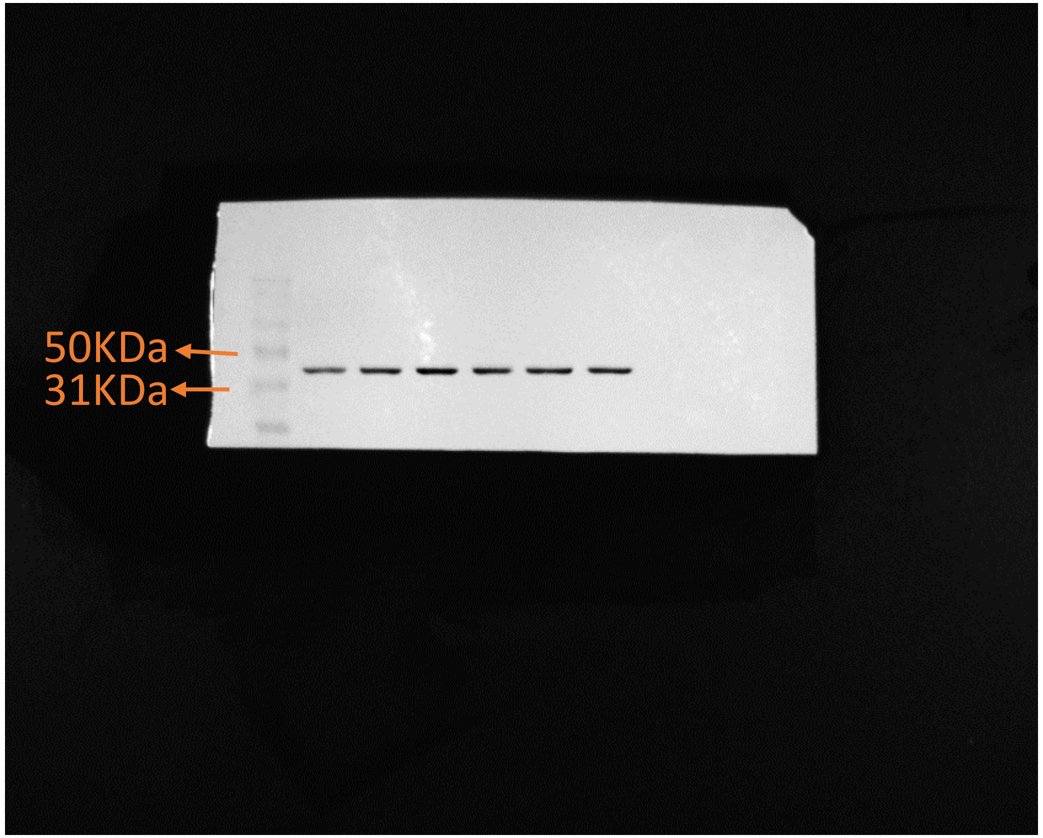


β-actin

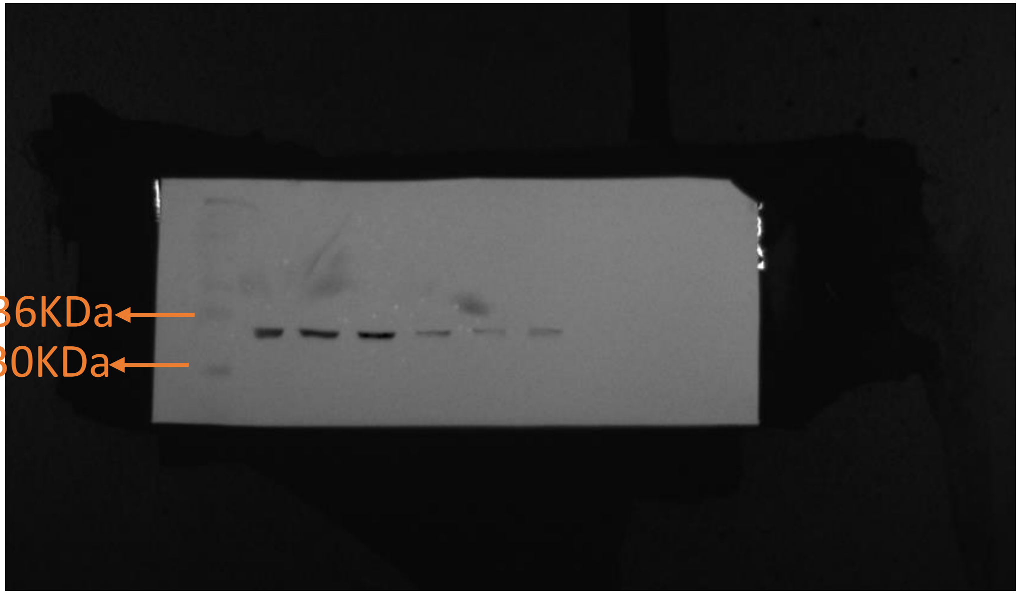


IKBα

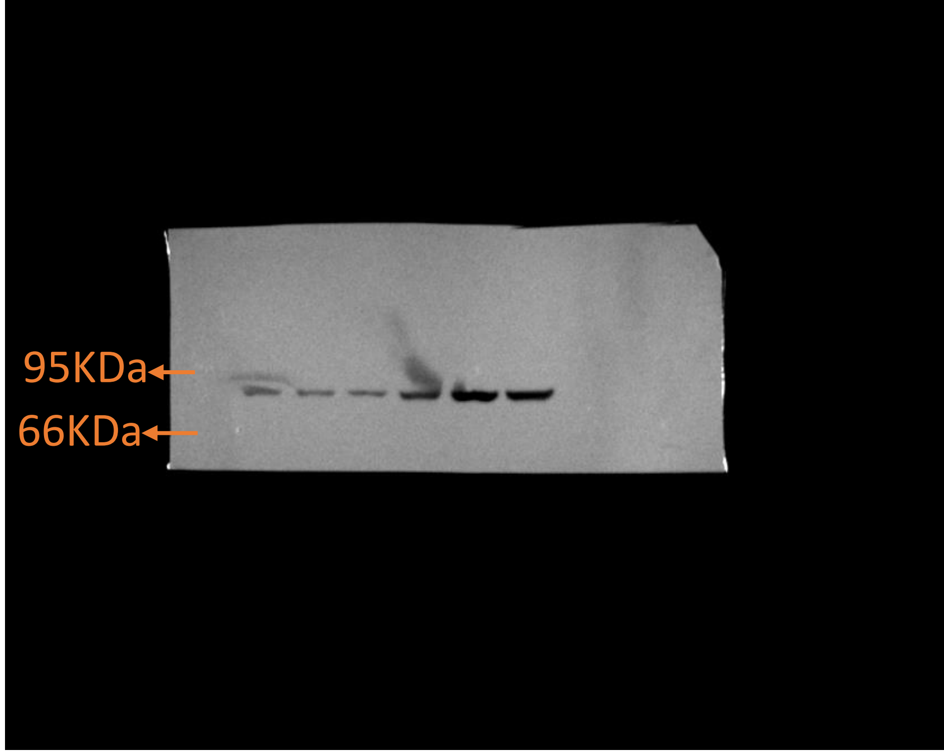


P-IKK

figure3.C Mouse liver

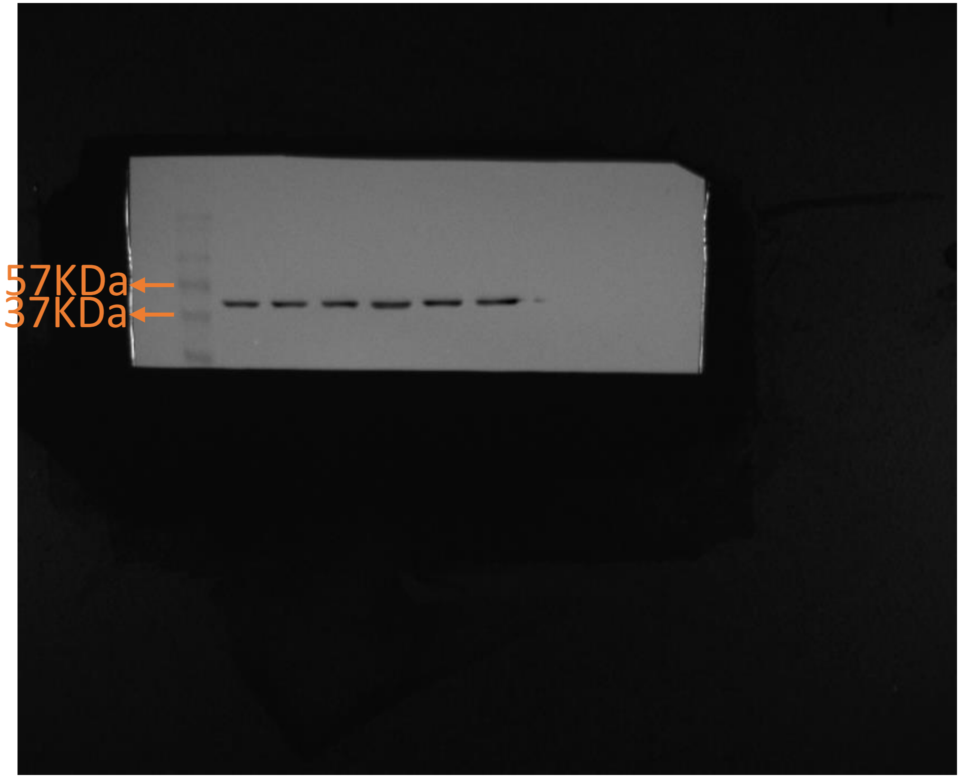


[illegible]

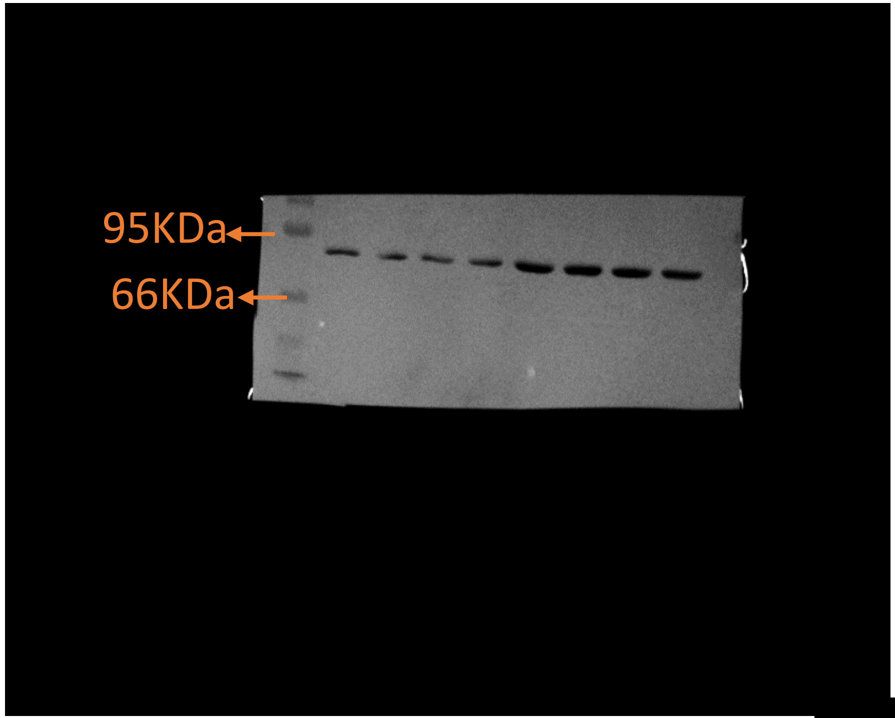

P-IKK

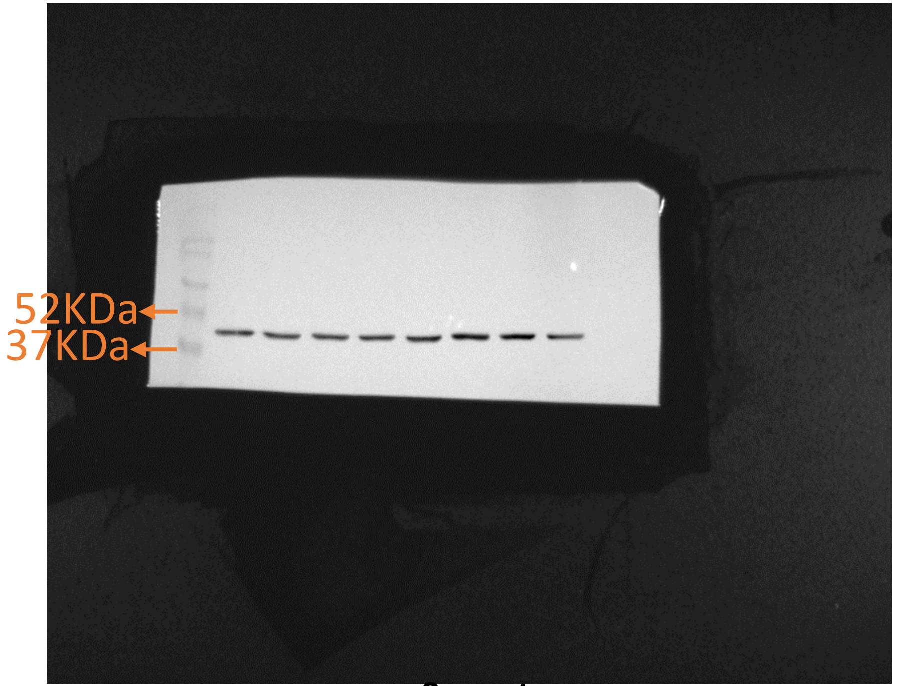

β-actin

figure3.D Mouse liver

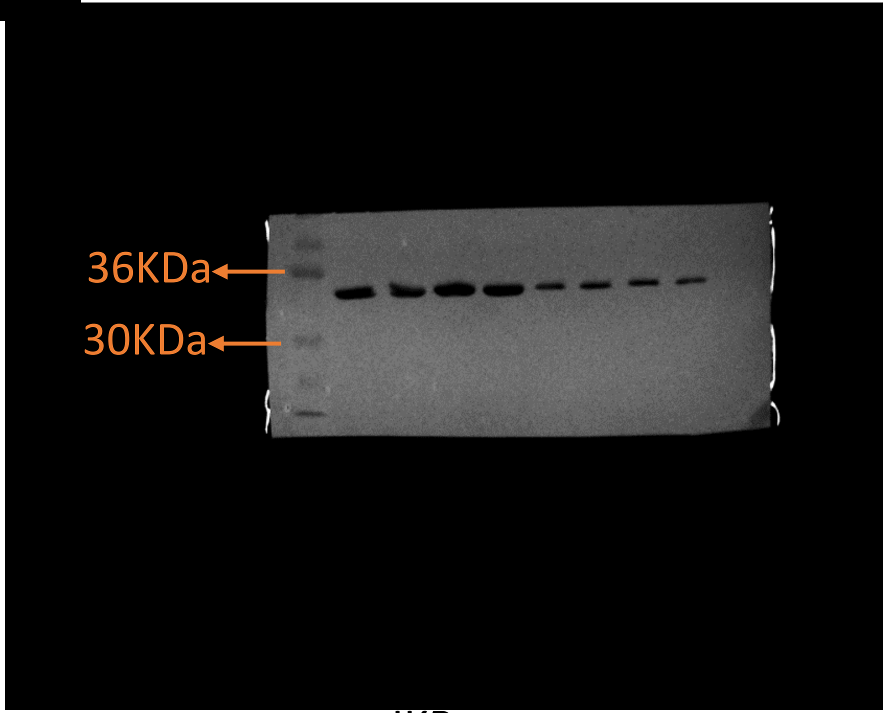

IKB-α

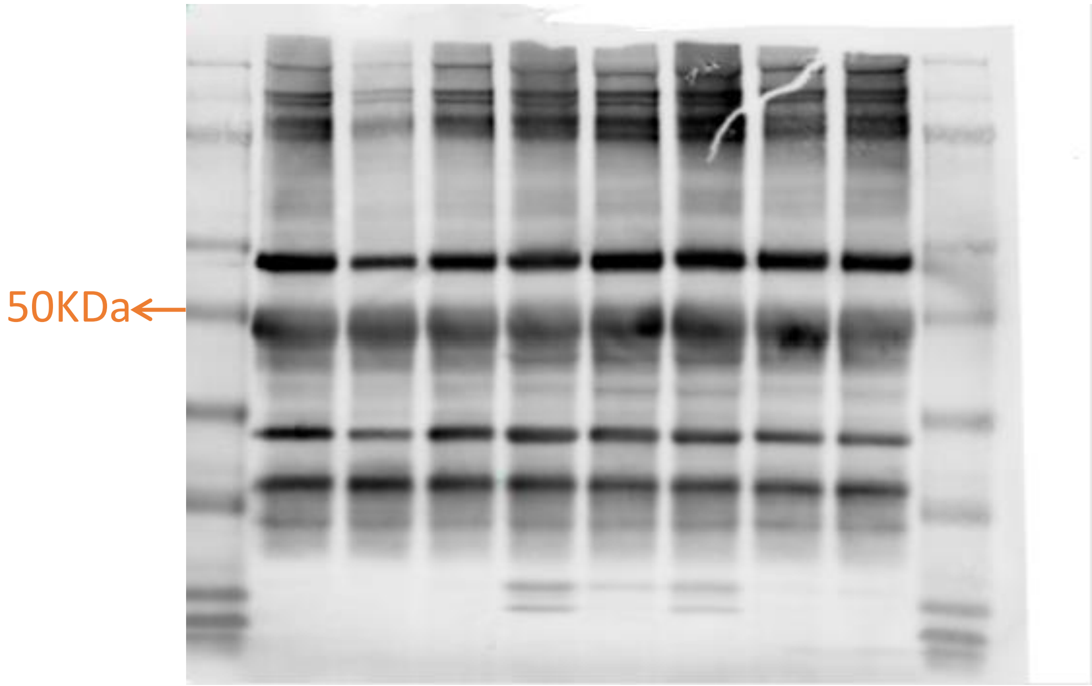


IP p65, WB p65

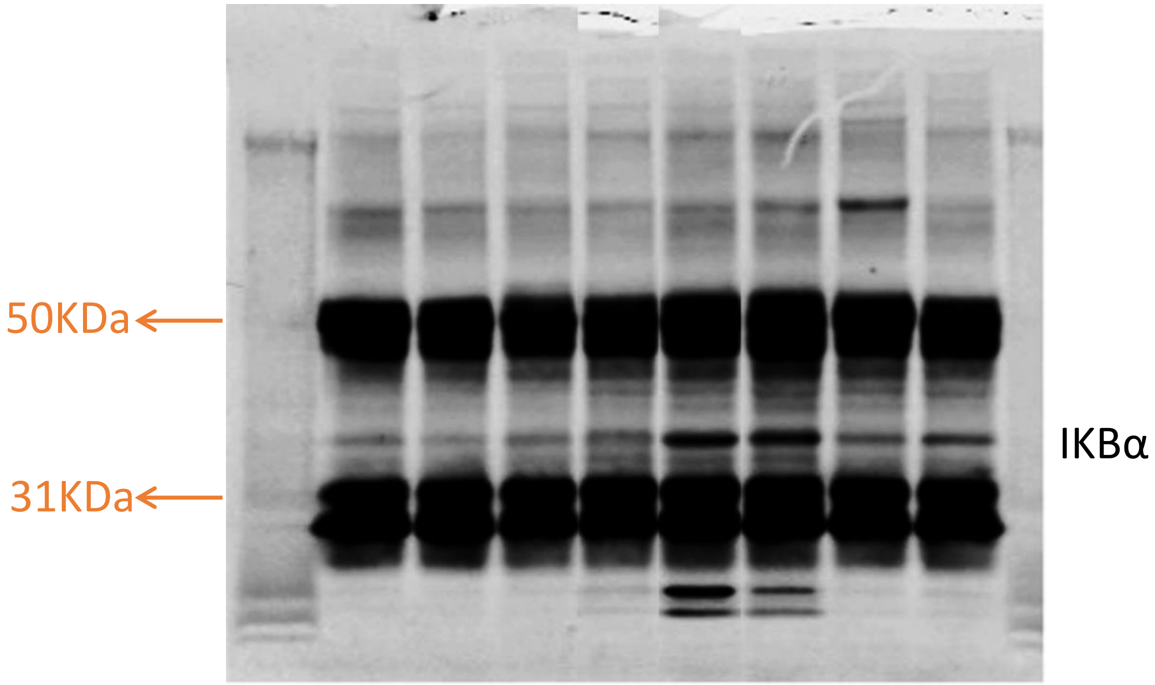


IP p65, WB IKBα

figure3.F Mouse liver

figure4.C Mouse liver

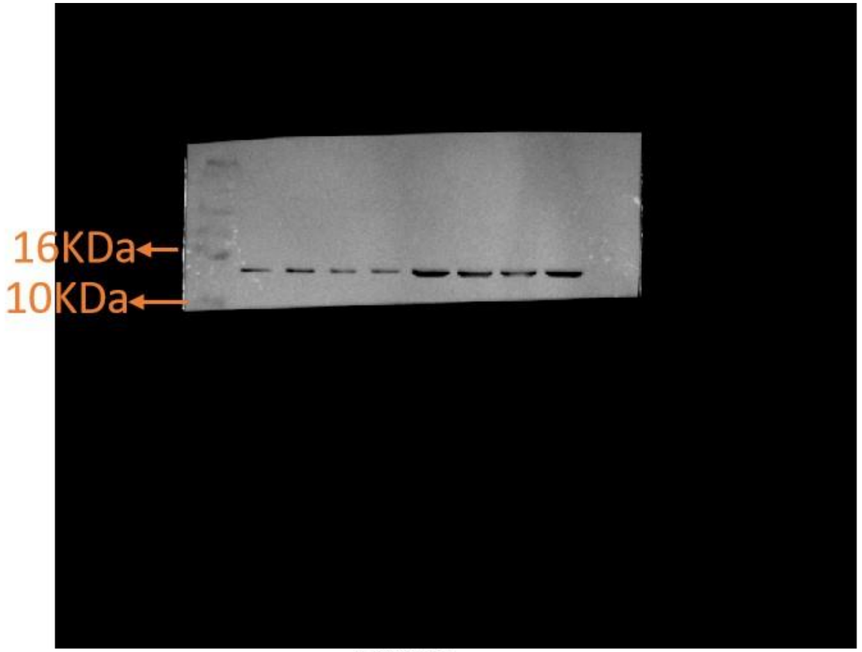


G0S2

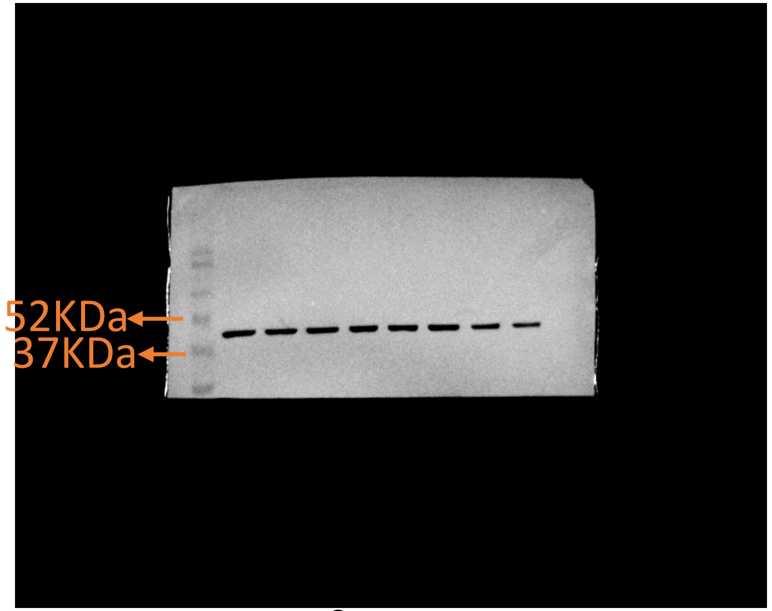


β-actin

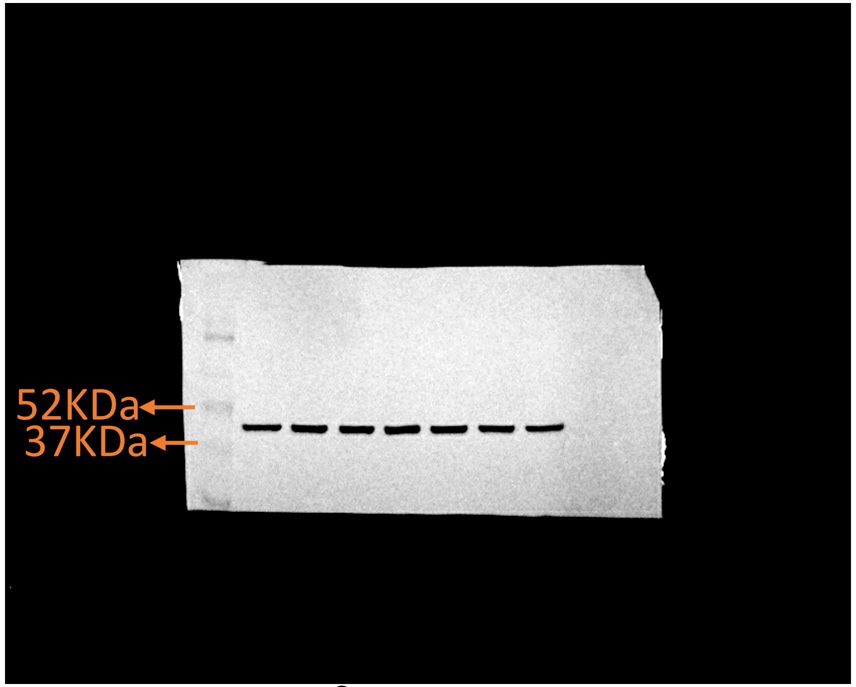


β-actin

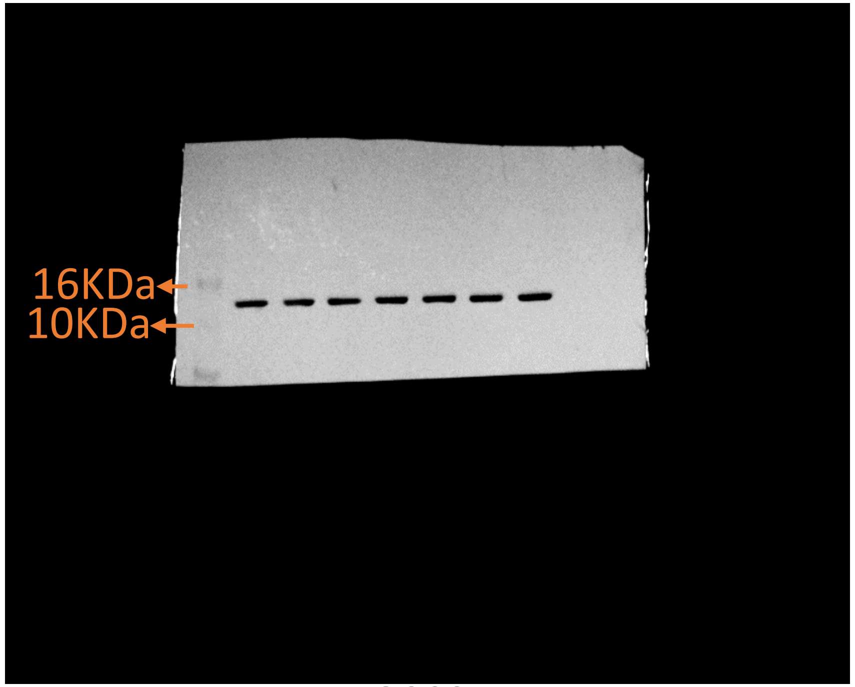


G0S2

figure4.D Mouse adipose tissue

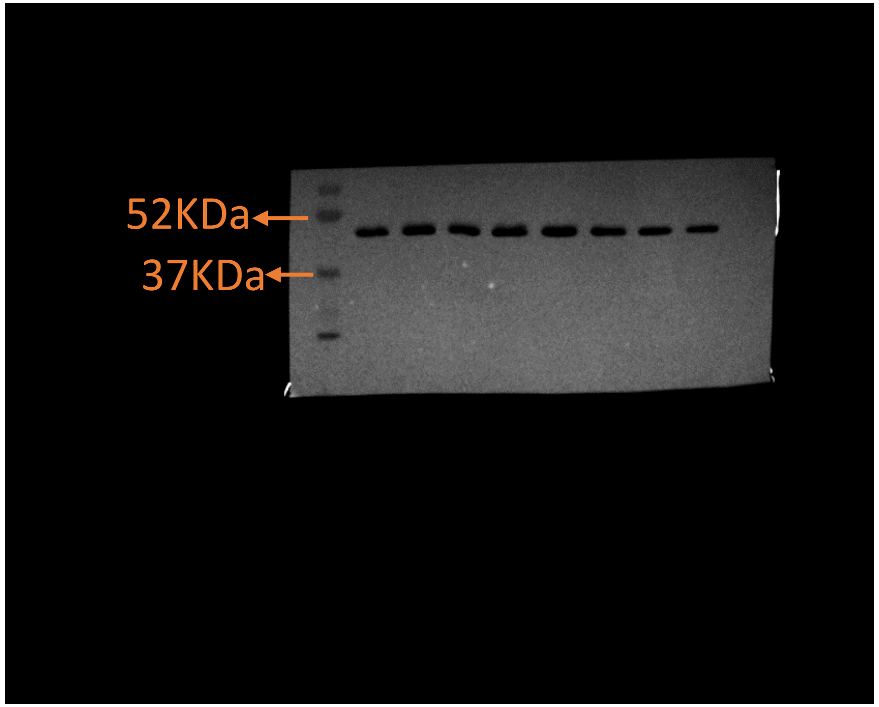

β-actin

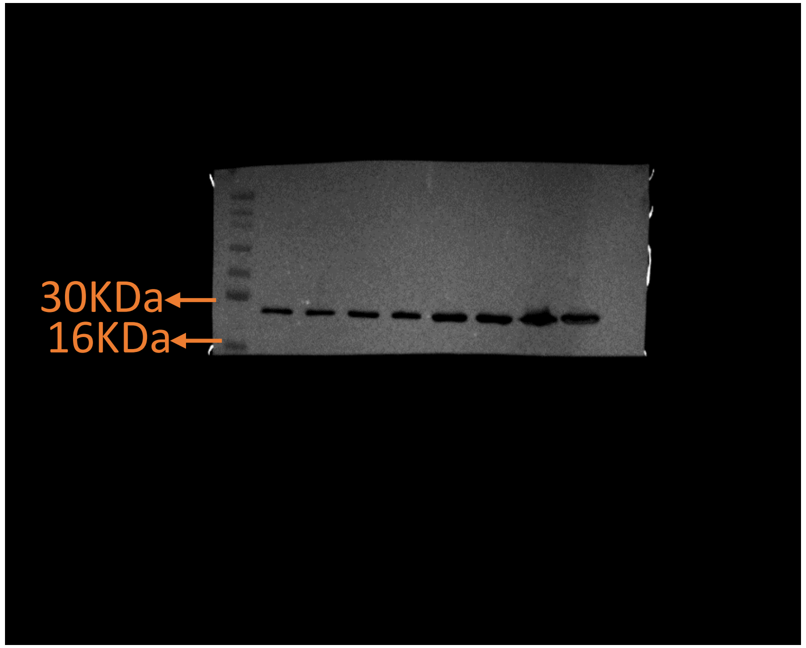

CHOP

figure4.F Mouse Liver

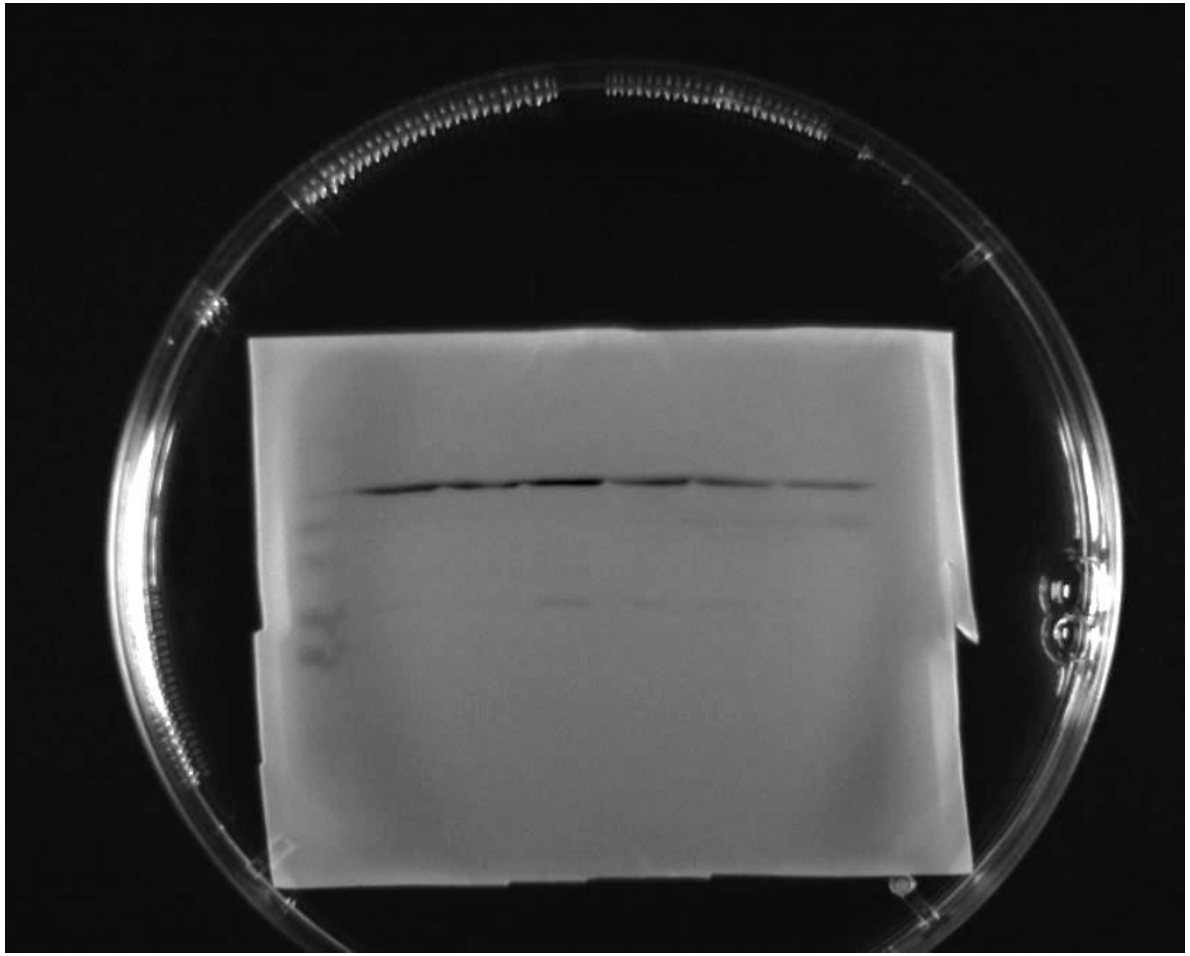

Supplementary Figure 3 A, IL-1β main band

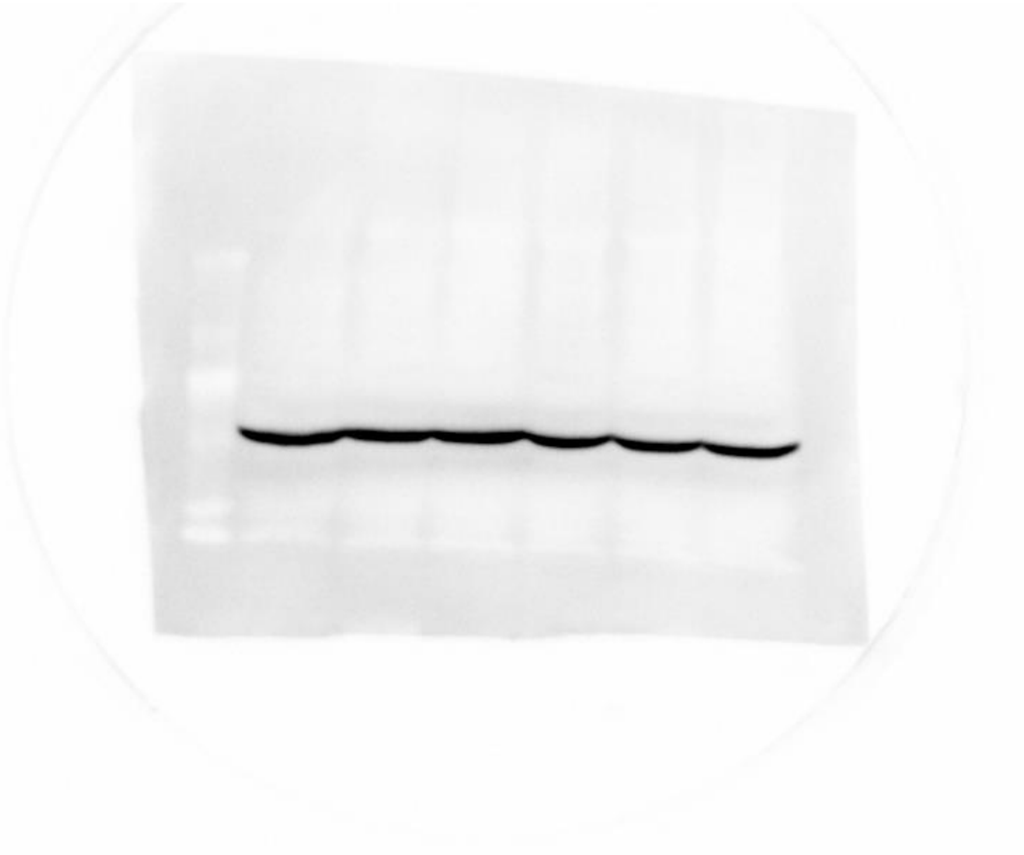

Supplementary Figure 3 A, β-actin

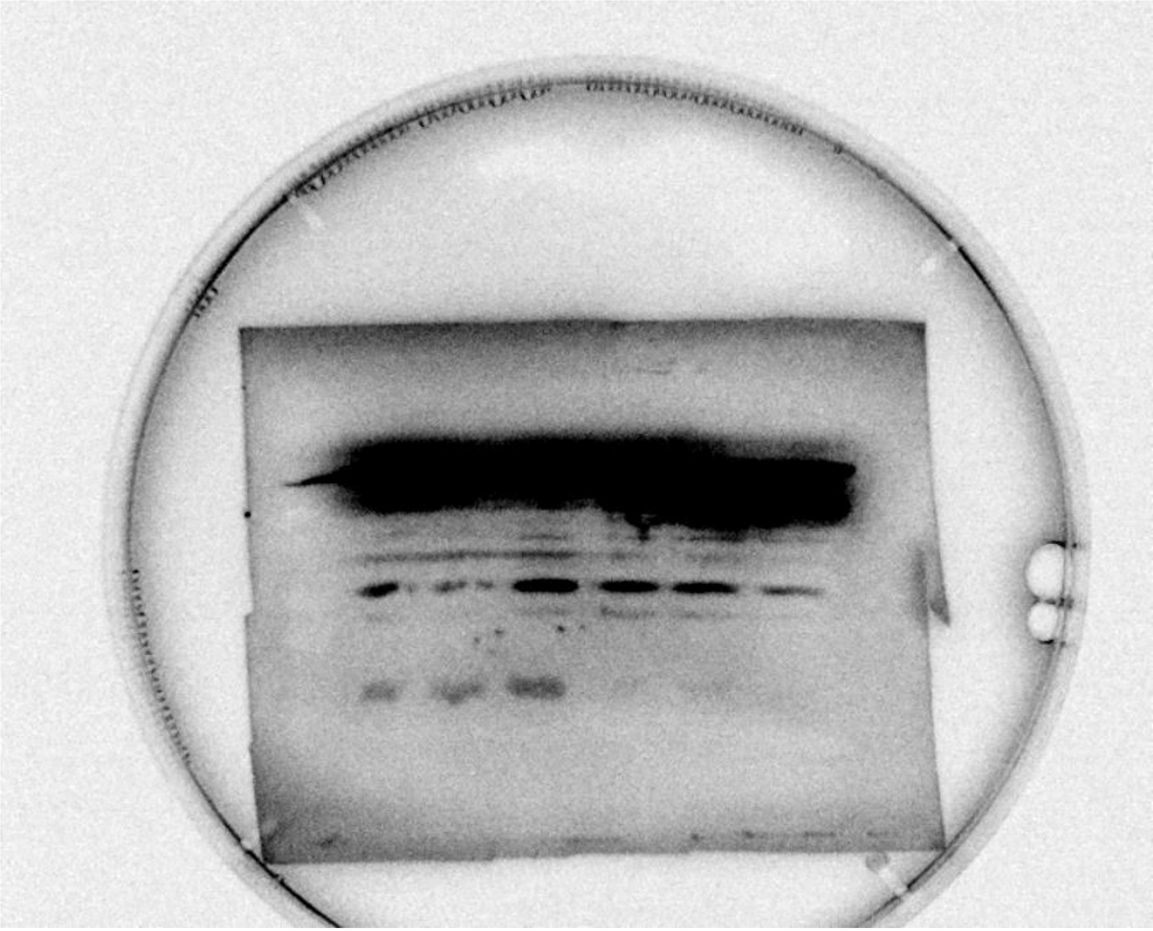

Supplementary Figure 3 C, IL-1β cleaved form

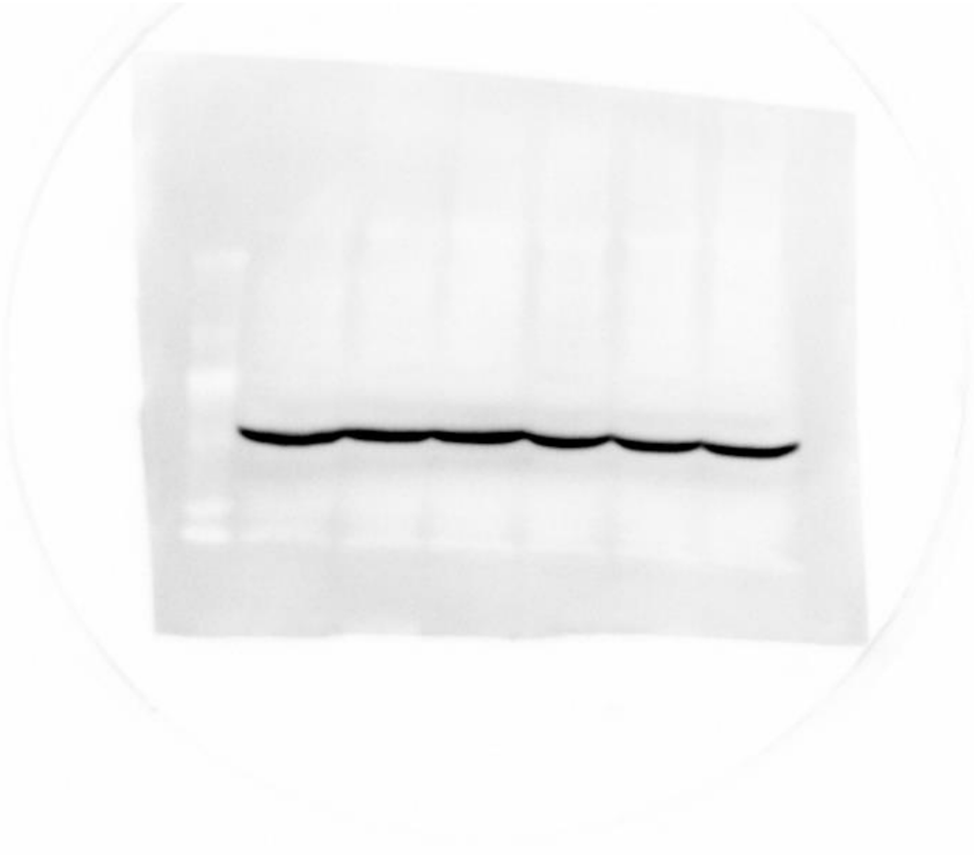

Supplementary Figure 3 C, β-actin

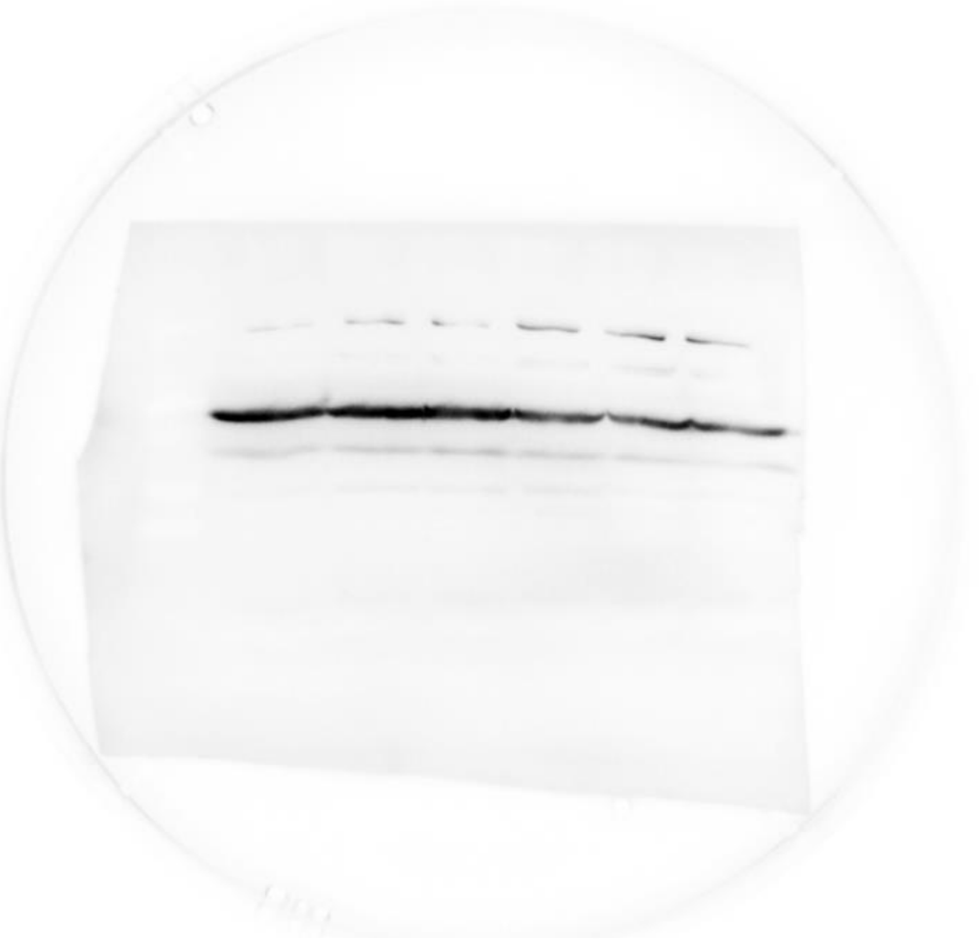

Supplementary Figure 3 D, Caspase-1 main band

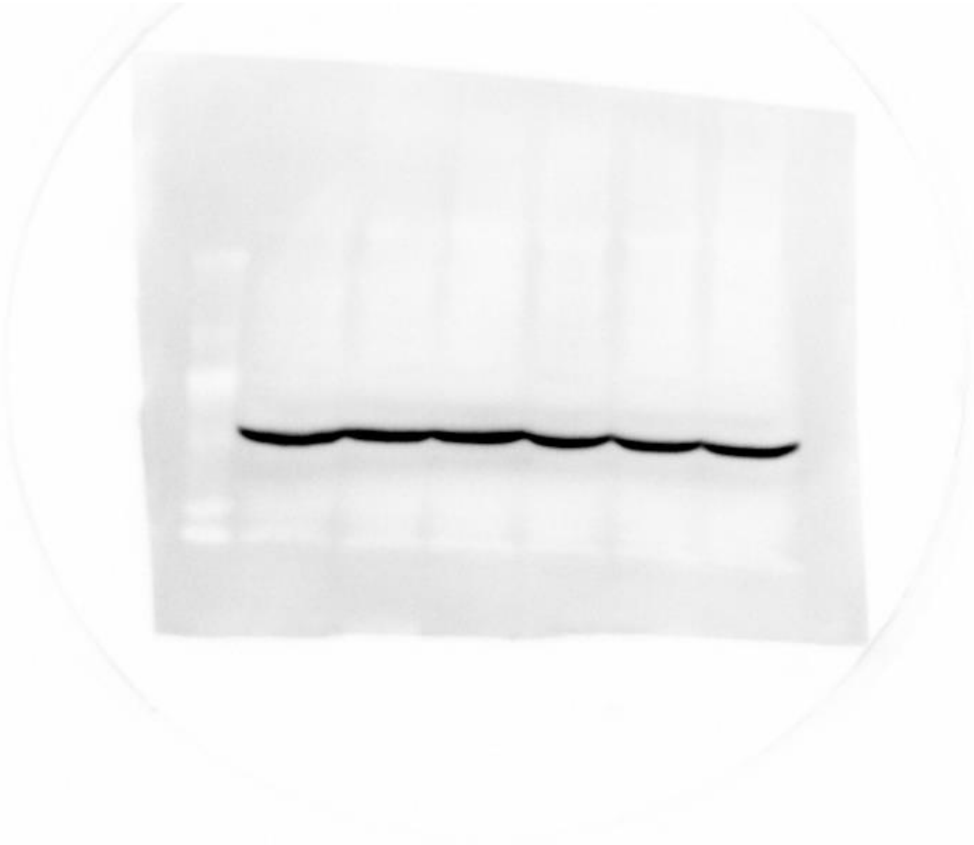

Supplementary Figure 3 D, β-actin

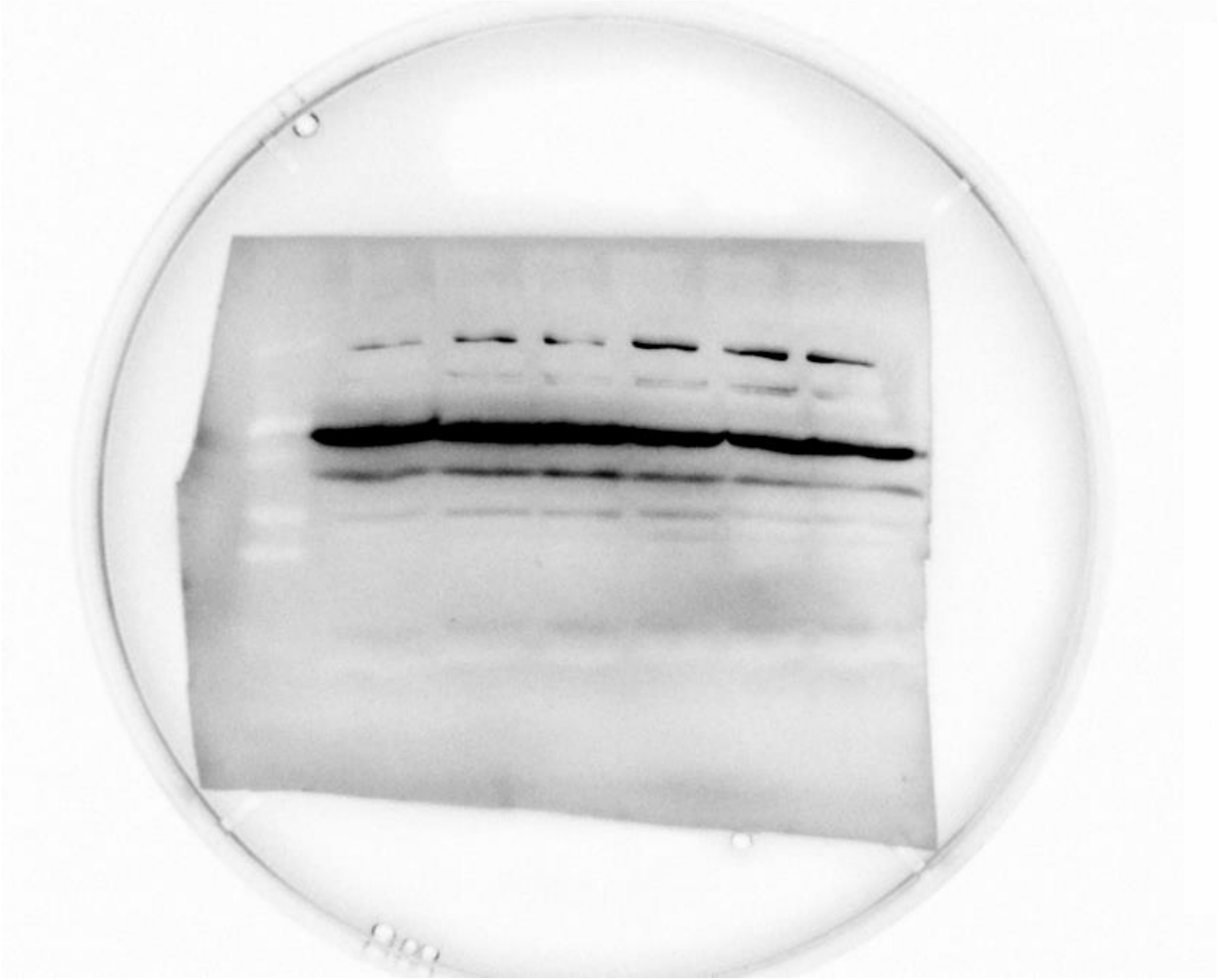

Supplementary Figure 3 F, cleaved Caspase-1

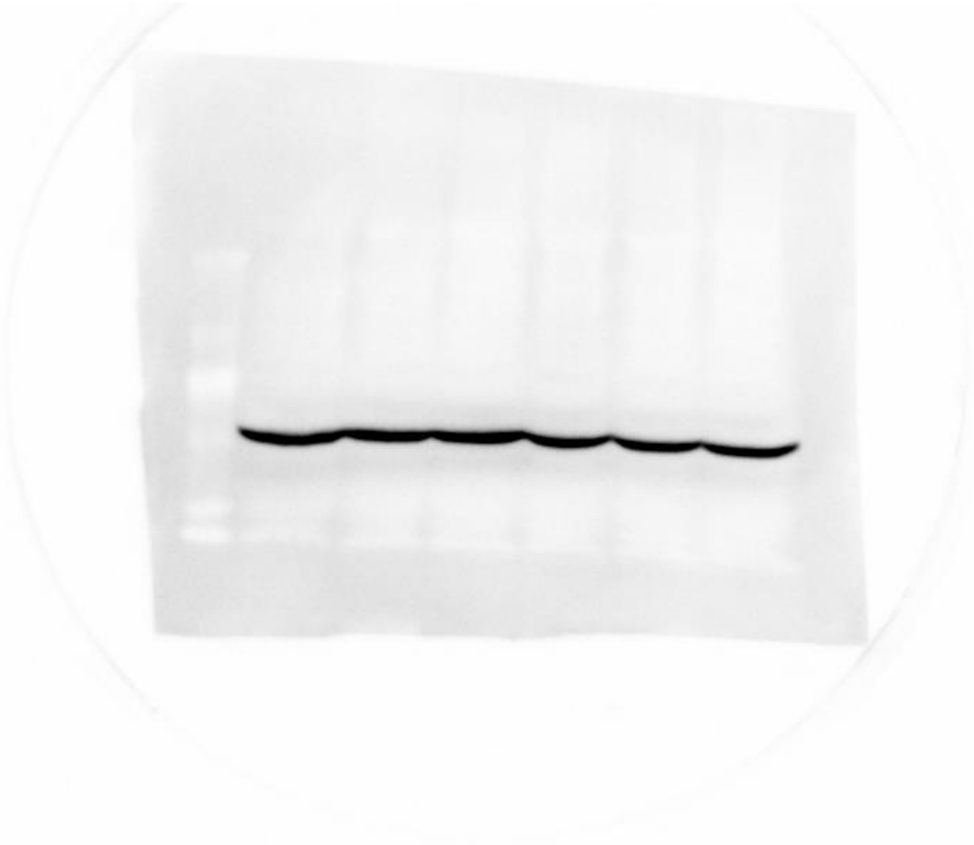

Supplementary Figure 3 F, β-actin
