# Supplementary material for: Rapamycin suppresses inflammation and increases the interaction between p65 and IκBα in rapamycin-induced fatty livers
Source: PLoS One. 2023 Mar 3;18(3):e0281888. doi: 10.1371/journal.pone.0281888 (PMC9983852; doi:10.1371/journal.pone.0281888)
Supplement: Attachment — Submitted filename: Response to reviewers.docx [file pone.0281888.s006.docx]

PONE-D-22-30305

Rapamycin suppresses inflammation and increases the interaction between p65 and IκBα in rapamycin-induced fatty liver

PLOS ONE

Dear Dr. Yu,

Thank you for submitting your manuscript to PLOS ONE. After careful consideration, we feel that it has merit but does not fully meet PLOS ONE’s publication criteria as it currently stands. Therefore, we invite you to submit a revised version of the manuscript that provides the clarifications requested by the reviewer.

Please submit your revised manuscript by Jan 16 2023 11:59PM. If you will need more time than this to complete your revisions, please reply to this message or contact the journal office at [plosone@plos.org](mailto:plosone@plos.org). When you're ready to submit your revision, log on to https://www.editorialmanager.com/pone/ and select the 'Submissions Needing Revision' folder to locate your manuscript file.

Please include the following items when submitting your revised manuscript:

- A rebuttal letter that responds to each point raised by the academic editor and reviewer(s). You should upload this letter as a separate file labeled 'Response to Reviewers'.
- A marked-up copy of your manuscript that highlights changes made to the original version. You should upload this as a separate file labeled 'Revised Manuscript with Track Changes'.
- An unmarked version of your revised paper without tracked changes. You should upload this as a separate file labeled 'Manuscript'.

If you would like to make changes to your financial disclosure, please include your updated statement in your cover letter. Guidelines for resubmitting your figure files are available below the reviewer comments at the end of this letter.

If applicable, we recommend that you deposit your laboratory protocols in protocols.io to enhance the reproducibility of your results. Protocols.io assigns your protocol its own identifier (DOI) so that it can be cited independently in the future. For instructions see: <https://journals.plos.org/plosone/s/submission-guidelines#loc-laboratory-protocols>. Additionally, PLOS ONE offers an option for publishing peer-reviewed Lab Protocol articles, which describe protocols hosted on protocols.io. Read more information on sharing protocols at <https://plos.org/protocols?utm_medium=editorial-email&utm_source=authorletters&utm_campaign=protocols>.

We look forward to receiving your revised manuscript.

Kind regards,

David M. Ojcius
Academic Editor
PLOS ONE

Journal requirements:

When submitting your revision, we need you to address these additional requirements.

1.  Please ensure that your manuscript meets PLOS ONE's style requirements, including those for file naming. The PLOS ONE style templates can be found at

<https://journals.plos.org/plosone/s/file?id=wjVg/PLOSOne_formatting_sample_main_body.pdf>  and

<https://journals.plos.org/plosone/s/file?id=ba62/PLOSOne_formatting_sample_title_authors_affiliations.pdf>

Response: We have modified our manuscript style according to your instructions!

2. PLOS ONE now requires that authors provide the original uncropped and unadjusted images underlying all blot or gel results reported in a submission’s figures or Supporting Information files. This policy and the journal’s other requirements for blot/gel reporting and figure preparation are described in detail at https://journals.plos.org/plosone/s/figures#loc-blot-and-gel-reporting-requirements and https://journals.plos.org/plosone/s/figures#loc-preparing-figures-from-image-files. When you submit your revised manuscript, please ensure that your figures adhere fully to these guidelines and provide the original underlying images for all blot or gel data reported in your submission. See the following link for instructions on providing the original image data: https://journals.plos.org/plosone/s/figures#loc-original-images-for-blots-and-gels.

In your cover letter, please note whether your blot/gel image data are in Supporting Information or posted at a public data repository, provide the repository URL if relevant, and provide specific details as to which raw blot/gel images, if any, are not available. Email us at plosone@plos.org if you have any questions.

Response: we would like to include all the original uncropped western blotting images in the Supporting Information. And we will indicate this point in our new cover letter.

3. To comply with PLOS ONE submissions requirements, in your Methods section, please provide additional information regarding the experiments involving animals and ensure you have included details on (1) methods of sacrifice, (2) methods of anesthesia and/or analgesia, and (3) efforts to alleviate suffering.

Response: we will include these pieces of information in our revised manuscript.

4. Thank you for stating the following in the Acknowledgments Section of your manuscript:

“This work was supported by the funding of Yunnan Key Laboratory for Basic Research on Bone and Joint Diseases (BRBJD-2021-1) and the funding of Kunming University (XJ20220008).”

We note that you have provided funding information that is currently declared in your Funding Statement. However, funding information should not appear in the Acknowledgments section or other areas of your manuscript. We will only publish funding information present in the Funding Statement section of the online submission form.

Please remove any funding-related text from the manuscript and let us know how you would like to update your Funding Statement. Currently, your Funding Statement reads as follows:

“This work was supported by the funding of Yunnan Key Laboratory for Basic Research on Bone and Joint Diseases (BRBJD-2021-1) and the funding of Kunming University (XJ20220008) awarded to An Yu. Yunnan Key Laboratory for Basic Research on Bone and Joint Diseases is a lab of Kunming University. The URL of Kunming University is https://www.kmu.edu.cn/  No, the sponsors did not play any role in the study design”

Please include your amended statements within your cover letter; we will change the online submission form on your behalf.

Response: we will remove funding information from the Acknowledgments section. Thank you for your instructions.


[Note: HTML markup is below. Please do not edit.]

Reviewers' comments:

Reviewer's Responses to Questions

**Comments to the Author**

1. Is the manuscript technically sound, and do the data support the conclusions?

The manuscript must describe a technically sound piece of scientific research with data that supports the conclusions. Experiments must have been conducted rigorously, with appropriate controls, replication, and sample sizes. The conclusions must be drawn appropriately based on the data presented.

Reviewer #1: Yes

2. Has the statistical analysis been performed appropriately and rigorously?

Reviewer #1: Yes

3. Have the authors made all data underlying the findings in their manuscript fully available?

The [PLOS Data policy](http://www.plosone.org/static/policies.action#sharing) requires authors to make all data underlying the findings described in their manuscript fully available without restriction, with rare exception (please refer to the Data Availability Statement in the manuscript PDF file). The data should be provided as part of the manuscript or its supporting information, or deposited to a public repository. For example, in addition to summary statistics, the data points behind means, medians and variance measures should be available. If there are restrictions on publicly sharing data—e.g. participant privacy or use of data from a third party—those must be specified.

Reviewer #1: Yes

4. Is the manuscript presented in an intelligible fashion and written in standard English?

PLOS ONE does not copyedit accepted manuscripts, so the language in submitted articles must be clear, correct, and unambiguous. Any typographical or grammatical errors should be corrected at revision, so please note any specific errors here.

Reviewer #1: No

5. Review Comments to the Author

Please use the space provided to explain your answers to the questions above. You may also include additional comments for the author, including concerns about dual publication, research ethics, or publication ethics. (Please upload your review as an attachment if it exceeds 20,000 characters)

Reviewer #1: The authors describe the effects of rapamycin in mice treated with rapamycin to induce fatty liver. The study appears to have been well performed and the conclusions are supported by the data. However, some questions came out during the review which requires clarifications and revisions.

Response: Dear reviewer, thank you very much for your careful work! We will try our best to address your concerns.

Detailed comments:
1) The design of the study is quite counter-intuitive. How can a compound like rapamycin help reduce a symptom or disease that it caused in the first place? Why would people take rapamycin as an anti-aging drug if it induces fatty liver disease, which can affect quality of life and increase toxicity to a wide range of other substances due to poor liver function? Can the dose of rapamycin be reduced so that it does not induce fatty liver disease? How can we confirm that the first disease-causing exposure to rapamycin did not affect the subsequent treatment phase in terms of metabolism/phamacokinetics of the drug and induced drug resistance? Is the study design a relevant scenario for humans? Inflammation also contributes to fatty liver disease so this can hardly described as fatty liver disease as seen also by the absence of fibrosis.

Response: Yes, this study is kind of counter-intuitive, however, the design of this study is based on what has been observed regarding the effects of rapamycin on mice and even on humans. On one hand, rapamycin consistently extends the lifespans of mice in several studies (PMID: 19587680, 33145977, 35796299, 36179270, 23863708, 27549339); on the other hand, rapamycin treatment did cause fatty liver in mice (PMID: 23562079), and this phenotype is consistent with what we have observed in our experiments (Fig. 1A&B). Rapamycin has been proposed to prevent liver fibrosis or liver cirrhosis (PMID: 19790156, 10535884, 24966615, 27315465, 35405287); usually, liver fibrosis or liver cirrhosis is a devastating consequence of fatty liver, especially, alcohol-induced fatty liver and high fat diet-induced non-alcohol fatty liver, but rapamycin indeed induces fatty liver at the first place. One reasonable explanation for this paradox is that rapamycin-induced fatty liver is quite different from alcohol-induced fatty liver and high fat diet-induced fatty liver. The current study aims to distinguish rapamycin-induced fatty liver from other types of fatty livers such as a high fat diet-induced fatty lever, and we found that although the increased lipid accumulation and increased free fatty acid levels were observed in rapamycin-induced fatty liver, the expression levels of pro-inflammatory genes are even lower than that in the control group. This result indicated that rapamycin-induced fatty liver indeed is different from high fat diet-induced fatty liver, at least in the aspects of inflammation and fibrosis (Fig. 4G). Rapamycin is an FDA-approved drug that can be used to suppress Immunol-rejection after organ transplantations, yet a few people take rapamycin as an anti-aging drug due to the fact that rapamycin shows many slowing aging effects in a lot of pre-clinic studies, even though these studies also showed many side effects including fatty liver. These side effects indeed restrict broader applications of rapamycin, however, as long as people can prove that the side effects caused by rapamycin are less harmful than that was previously thought, or other drugs can cancel/reduce the side effects caused by rapamycin, then rapamycin might be considered a safer anti-aging drug that can be broadly used in the future. In addition, rapamycin does not necessarily cause side effects in all persons who use it, just like penicillin which saved many people’s lives from bacterial infections though it could be dangerous to people who are allergic to it. The key determinant of whether rapamycin can be broadly used as an anti-aging drug is if the anti-aging beneficial effects are much more than the detrimental side effects. One goal of the current study is to explain that fatty liver caused by rapamycin is less harmful than that was previously thought because rapamycin-induced fatty liver did not show increased inflammation levels (Figure 3 A&E) and did not increase the expression levels of fibrosis factors (Figure 4 G). The dose of rapamycin we used in this study is very similar to that used before, which has been proven competent to extend mouse lifespans (PMID: 20974732). If we reduced the rapamycin dose, which might be helpful to reduce fatty liver, we are not sure if the life-extending effect can be preserved due to the reduced dose. We cannot confirm that the first disease-causing exposure to rapamycin did not affect the subsequent treatment phase, and we also cannot exclude the effects of drug resistance, but as long as the final outcome of rapamycin treatment shows more beneficial effects than the adverse effects, then this drug is worthy to have more investigations in the future. Considering many studies in mice showed the life-extending effects of rapamycin, we assume that the final outcome of rapamycin treatment is a good one. The results of this study can be used as a reference for humans, but the results only reflect the situation of rapamycin treatment in mice; if we can gain funding with more money, we would happily like to organize a clinical trial referred to rapamycin side effects in the future. Yes, we can hardly term rapamycin-induced fatty liver as a disease, because it is more likely not to develop into liver fibrosis (Fig.4G) as other types of the fatty liver did, instead, we would more like to call it a symptom.

2) What is the relevance for human use of injecting rapamycin intra-peritoneally? Does rapamycin induce liver disease orally?

Response: The reason why we used intra-peritoneally injection of rapamycin in mice is that we can give each mouse rapamycin in an accurate dosage. If we mixed rapamycin in the chow diet, then the rapamycin amount that was received by each mouse will be variable due to the different food amounts that were taken by each mouse from day to day. Indeed, orally taking rapamycin caused dyslipidemia (PMID: 24654608) in some patients, and considering the liver is a major contributor to triglyceride levels in the blood, we assume that this result may imply that orally taking rapamycin in humans did affect lipid metabolism in the liver.

3) Rapamycin does mimic some aspects of fasting, mainly due to its capacity to inhibit mTOR which is activated by food, especially proteins. The text lacks this kind of nuances in the description of the results.
Response: Indeed, both fasting and rapamycin treatment are able to inhibit mTOR activity (PMID: 31406105), therefore rapamycin does mimic some aspects of fasting; however, in our current study, we found that rapamycin treatment and 24 h fasting induced very different gene expression patterns (Fig.1C). The reasons behind this phenomenon, we thought, are that rapamycin mainly affects mTOR signaling whereas fasting affects many pathways including Sirts (Sirt1-Sirt7), AMPK, and mTOR signaling. We will add these descriptions to the text.

4) In section 3.2, the authors conclude that rapamycin reduced mRNA levels of the NLRP3 inflammasome, but the results are incomplete since a two-hit mechanism is needed to induce its activation and cleavage of caspase-1 and IL-1beta. Further experiments are needed to make a conclusion about the effects on the NLRP inflammasome. For instance, the levels of cleaved caspase-1 and IL-1beta protein should be examined.

Response: We purchased Caspase-1 (cat# ab179515) and IL-1β (cat# ab234437) antibodies from Abcam and new western blotting experiments were added. The results of these western blotting experiments were put in Supplementary Figure 3. As has been shown in Supplementary Figure 3, 8-day rapamycin treatment significantly decreased IL-1β protein levels in mouse livers (Supplementary Figure 3 A&B), and notably, the cleaved IL-1β for secretion was hardly detectable in rapamycin-induced fatty livers, though another cleaved IL-1β protein levels were not affected by rapamycin (Supplementary Figure 3 C). Also, rapamycin treatment significantly decreased Caspase-1 protein levels (Supplementary Figure 3 D, E&F), though it is hard to determine whether cleaved Caspase-1 levels were affected by rapamycin due to the very low signaling (Supplementary Figure 3 F). These results indicate that NLRP3 inflammasome activation may be suppressed by rapamycin at least via decreasing Caspase-1 and IL-1β expression levels and reducing cleaved IL-1β form for secretion.

5) Previous studies have shown that free fatty acids can induce inflammation. In Fig. 2F, the authors show that rapamycin increases fatty acids in the liver, without inducing inflammation. The authors should present possible reasons to explain this paradox in the text.

Response: our experimental results indicated that although increased free fatty acid levels were observed (Fig. 2F) and the upper stream of the NFκB pathway was activated (Fig. 3D) in rapamycin-induced fatty livers, the translocation of p65 into cellular nuclear was not increased (Fig. 3E), preventing that NFκB functions as a pro-inflammatory transcriptional factor. This is one of the reasonable explanations for the paradox. We would like to emphasize this point in the DISCUSSION part.

6) The discussion should be re-written to present more insights from the results, comparisons with previous studies, possible limitations of the study, implications of the results, and/or future questions to be addressed.
Response: we have rewritten the discussion according to the reviewer’s suggestions.

7) Scale bars are missing on the microscopy images.

Response: Scale bars have been added now.

8) Some sentences should be rewritten for clarity. Grammar and typos should be checked throughout.

Response: We have had our manuscript checked and edited by an English native speaker.
